# Effect of Zinc and Vitamin E on Blood Testosterone and Inflammatory Markers in Male Patients Undergoing Heart Surgery

**DOI:** 10.5812/ijem-147892

**Published:** 2024-08-07

**Authors:** Javad Nasrollahzadeh, Elham Makiabadi, Mohammad Reza Shahparvari, Maryam Nilghaz, Behnaz Narimani, Yegane Rajabpour Ranjbar

**Affiliations:** 1 Department of Clinical Nutrition and Dietetics, National Nutrition and Food Technology Research Institute, Faculty of Nutrition Sciences and Food Technology, Shahid Beheshti University of Medical Sciences, Tehran, Iran

**Keywords:** Zinc, Vitamin E, Testosterone, Toll-Like Receptor, Coronary Artery Bypass Graft, Interleukin 6

## Abstract

**Background:**

Zinc and vitamin E affect the metabolism of testosterone and inflammatory factors. We aimed to evaluate the effect of zinc and vitamin E supplementation on plasma testosterone levels and inflammatory markers in patients undergoing coronary artery bypass graft (CABG) surgery.

**Methods:**

This study was a secondary analysis of a previously published randomized controlled trial in a subsample of male patients undergoing CABG surgery. Patients in the zinc-vitamin E group (n = 27) received oral zinc (120 mg) and vitamin E (1200 international units) one day prior to surgery, followed by 30 mg of zinc and 200 units of vitamin E per day for three weeks after surgery. Patients in the control group (n = 25) received a placebo. Plasma levels of total testosterone, cortisol, interleukin-6 (IL-6), and white blood cell toll-like receptor-4 (TLR-4) gene expression were determined at three-day and three-week intervals following surgery. Changes in these markers were analyzed using a repeated-measures analysis of variance.

**Results:**

A comparison of the groups revealed no significant difference in the concentration of plasma total testosterone levels (P = 0.059) or cortisol. Three weeks following the surgical procedure, a positive correlation was observed between the change in plasma zinc concentrations and the change in plasma testosterone levels (r = 0.32; P = 0.025). The administration of zinc and vitamin E supplements resulted in a reduction in plasma IL-6 levels on postoperative day 3 (P = 0.025), while no significant effect was observed in week 3 (P = 0.091). The expression of the TLR-4 gene in WBCs was found to be lower in the zinc-vitamin E group compared to the placebo group on day 3 (P = 0.051) and week 3 (P = 0.025).

**Conclusions:**

The administration of zinc and vitamin E to patients undergoing CABG was associated with a relative improvement in postoperative inflammatory markers. Plasma zinc levels demonstrated a correlation with testosterone levels, suggesting a potential avenue for further research in these patients.

## 1. Background

It has been demonstrated that major surgical procedures, such as coronary artery bypass grafting (CABG) using cardiopulmonary bypass, can induce an inflammatory response (1). Moreover, CABG is associated with substantial physiological stress and consequent changes in the endocrine systems. Following CABG surgery, there is a decline in circulating levels of anabolic hormones, such as testosterone (2, 3). This decline, coupled with an increase in catabolic hormones, such as cortisol (3), may result in a hormonal imbalance in the initial days following surgery. This, in conjunction with the rise in inflammatory markers, may contribute to complications post-surgery. Testosterone has been demonstrated to play a role in regulating inflammatory immune responses, as evidenced by studies (4, 5). A low testosterone concentration in critically ill male patients has been inversely related to the score of acute physiological and chronic health assessment (6). Moreover, a correlation between low testosterone levels and the necessity for mechanical ventilation due to acute respiratory failure has been documented in critically ill male patients (7). Furthermore, considering the anabolic role of testosterone, improving testosterone levels may assist in preventing lean mass loss and muscle dysfunction in postoperative or intensive care patients (8).

A correlation has been identified between low serum zinc concentrations and low serum testosterone levels in male subjects (9). The administration of oral doses of zinc supplements (50-57 mg of elemental zinc) to hemodialysis patients (10, 11) or to patients with sickle cell anemia (45 mg of elemental zinc) (12) has been observed to increase serum testosterone levels. In addition to zinc, vitamin E may also affect the synthesis or clearance of androgens (13). Moreover, zinc and vitamin E can also influence inflammatory responses. In healthy elderly individuals, oral administration of a zinc supplement (45 mg per day) has been shown to reduce the concentration of inflammatory factors (14). Similarly, in diabetic patients, oral administration of an alpha-tocopherol vitamin E supplement (1200 units per day) has been demonstrated to have a similar effect (15). The anti-inflammatory effect of these nutrients may be mediated through different mechanisms, including their effect on some receptors that identify specific molecular patterns associated with endogenous damage (16). Endogenous non-infectious danger signals that are ligands for toll-like receptors may be released during CABG surgery and cause the activation of innate immunity, thereby mediating the inflammatory response (17). A preclinical study has demonstrated that zinc administration reduces the expression of the toll-like receptor-4 (TLR-4) gene and the downstream signals associated with inflammation in weaning piglets (18).

## 2. Objectives

Previous studies have indicated the potential impact of zinc or vitamin E on testosterone concentration and inflammatory factors. In the present study, we postulated that perioperative zinc and vitamin E supplementation would influence postoperative plasma testosterone levels and modulate the expression of TLR-4 in white blood cells in patients undergoing coronary artery bypass graft (CABG) surgery. Given the potential influence of sex on testosterone concentration, this study was limited to male patients.

## 3. Methods

### 3.1. Study Population

This study represents a secondary analysis of a previously published randomized, placebo-controlled trial (19) in a subsample of male patients undergoing CABG surgery. The methodology employed in this study has been previously described in detail. In summary, the study included patients scheduled for CABG surgery using the on-pump method who did not have liver cirrhosis, end-stage kidney disease, active cancer, chronic rheumatic diseases, or severe infection. Patients who regularly consumed micronutrient supplements or took corticosteroids and nonsteroidal anti-inflammatory drugs were excluded. Patients were randomly assigned to receive either zinc-vitamin E supplements or a placebo. In the zinc-vitamin E group, patients received oral zinc (120 mg elemental as zinc gluconate) and vitamin E (1200 international units) one day prior to surgery, and from the second day after surgery until three weeks later, they received 30 mg zinc and 200 units of vitamin E per day. In the placebo group, patients received placebo pearls. The study was conducted in accordance with the principles set forth in the Declaration of Helsinki and was approved by the ethics committee of the National Nutrition and Food Technology Research Institute. Prior to the commencement of the study, informed consent was obtained from all patients. The trial was registered prior to the enrollment of patients (NCT05402826).

### 3.2. Dietary Intake

The intake of food was evaluated using a 24-hour food recall at the outset of the study and in the final week of the study. Each time, the 24-hour recall was conducted twice, once on weekdays and once on weekends. The data were analyzed using Nutritionist 4 software (N-Squared Computing, USA).

### 3.3. Blood Sample Measurement

Venous blood samples were collected from patients in tubes containing heparin before surgery (the baseline) and on day 3 and week 3 after surgery. The samples were centrifuged, and plasma as well as buffy coat fractions were separated. Both plasma and buffy coats were stored at −80°C until analysis. The experiments were conducted by an experienced laboratory technician. Commercially available enzyme-linked immunosorbent assay kits were utilized for the quantification of plasma interleukin-6 (IL-6) (Biolegend, San Diego, USA), total testosterone, and cortisol (Monobind, Inc., Lake Forest, CA, USA).

### 3.4. RNA Extraction and Gene Expression Quantification

Total RNA was extracted from buffy coats using TRIzol reagent (Yekta Tajhiz Azma, Iran), and the purity and concentration of the extracted RNA were measured by NanoDrop 2000 (Thermo Scientific). Complementary DNA was synthesized from the extracted RNA using a commercially available reverse transcriptase kit (Pars Tous Biotech, Mashhad, Iran). Quantitative real-time polymerase chain reaction (qPCR) was employed to quantify the expression of toll-like receptor 4 (TLR-4) and the housekeeping gene glyceraldehyde-3-phosphate dehydrogenase (GAPDH). The qPCR was conducted using gene-specific primers and a SYBR green kit with ROX as a reference dye (qPCRBIOSyGreen Mix Hi-ROX, PCR Biosystem, UK). The primers utilized were as follows: TLR-4: forward primer: 5’-GCTCACAACCATCCTGGTCATT-3’; reverse primer: 5’-TTTGAAGCACGTCTTAAACAACCTTA-3’; GAPDH: Forward primer: 5’-CCAGGGCTGCTTTTAACTCT-3’; reverse primer: 5’-TGACAAGCTTCCCGTTCTCAG-3’. All reactions were conducted on a Step-One Plus system (Applied Biosystems) with a final volume of 20 µL. The thermal profile consisted of a 95°C incubation for two minutes, followed by 36 cycles of 95°C for five seconds and 60°C for 30 seconds, and finally a melting analysis. The delta delta Ct (ΔΔCt) values of the placebo groups were compared to the ΔΔCt values of the zinc-vitamin E groups. The ΔCt was calculated as Ct TLR-4 – Ct GAPDH, while the ΔΔCt was calculated as ΔCt at baseline – ΔCt on postoperative day 3 or week 3.

### 3.5. Statistical Analysis

In the original trial, the sample size was estimated based on the assumption that the intensive care unit length of stay would serve as the primary outcome, with an α = 5% and 95% power. The original trial recruited 78 patients (40 in the zinc-vitamin E group and 38 in the placebo group), with 27 patients in the zinc-vitamin E group and 25 patients in the placebo group identified as male. The Statistical Package for the Social Sciences (SPSS, version 26) was employed for the purpose of statistical analysis. The baseline characteristics of the study groups were compared using either the independent t-test (for normally distributed continuous variables) or the Mann-Whitney test (for skewed variables). Categorical variables were analyzed using the chi-square test. The changes in the studied markers were analyzed using repeated measures analysis of variance (ANOVA), with potential confounding variables included as covariates. Models were adjusted for the baseline value of each variable and the baseline body weight of patients, which differed between the two groups. To compare values between groups on days 3 and week 3 after surgery, univariate ANOVA was employed, with adjustments made for baseline values and body weight. The values of plasma zinc and IL-6 were not normally distributed and were therefore transformed using the log function prior to analysis to stabilize the variance. The white blood cells TLR-4 gene expression on postoperative days 3 and week 3 was analyzed using univariate analysis of covariance, with ΔΔCt entered as the dependent variable and body weight as a covariate. A correlation analysis was conducted on change values, defined as the difference between the values observed on day 3 or week 3 and the baseline values.

## 4. Results

**Figure 1.**
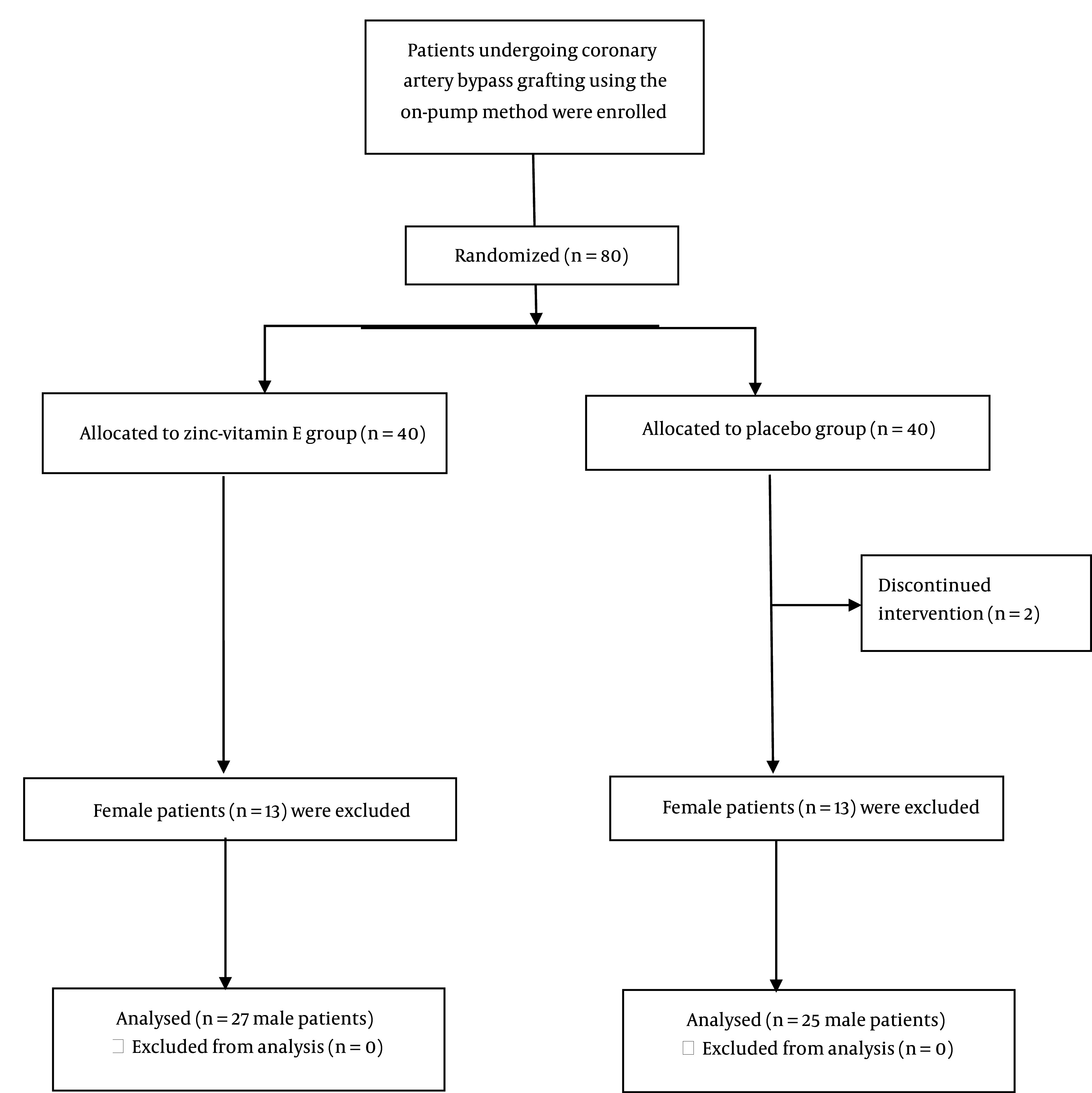
Flowchart of the study

A total of 13 patients (all women) from the zinc-vitamin E group and 13 patients (all women) from the placebo group were excluded from this study. The final analysis was conducted on 27 male patients from the zinc-vitamin E group and 25 male patients from the placebo group (Figure 1). Table 1 depicts the baseline characteristics of the patients. With the exception of body weight, baseline characteristics did not differ between the two groups. Table 2 shows the body weight and physical activity levels of the patients at the baseline and the end of the study. The body weight at the beginning and the third week after surgery was lower in the placebo group than in the zinc-vitamin E group. Both groups demonstrated a reduction in body weight following surgery. However, the placebo group exhibited a more pronounced decline than the zinc-vitamin E group. The level of physical activity, as determined by the physical activity questionnaire, remained unchanged between the two groups at both the baseline and the end of the study.

**Table 1.** Demographic and Baseline Clinical Characteristics of the Studied Patients in the Two Groups ^a^

Variables	Placebo (n = 25)	Zinc-Vitamin E Supplement (n = 27)	P ^b^
**Preoperative characteristics**			
Age (y)	58.0 (52.0, 71.0)	56.0 (51.0, 65.0)	0.25
Weight (kg)	64.3 ± 11.9	71.2 ± 12.4	0.049
BMI (kg/m^2^)	22.9 ± 3.7	24.5 ± 4.1	0.14
History of smoking	3 (12)	6 (22)	0.33
**Comorbidities**			
Diabetes	5 (20)	5 (18.5)	0.90
Dyslipidemia	3 (12)	6 (22)	0.33
Hypertension	9 (36)	13 (48)	0.37
**Preoperative blood values**			
Creatinine (mg/dL)	1.2 (0.9, 1.3)	1.0 (0.9, 1.2)	0.33
Albumin (g/dL)	3.9 (3.5, 4.4)	4.3 (3.9, 4.4)	0.25
Zinc (µg/dL)	61.0 (48.5, 72)	61.0 (50.0, 66.0)	0.82
WBC (10^3^/mm^3^)	8.0 (6.0, 9.6)	6.8 (6.1, 8.8)	0.20
ESR (mm/h)	12.0 (10.0, 31.0)	13.0 (6.0, 28.0)	0.61
CRP (mg/L)	1.4 (0.4, 2.3)	2.64 (0.9, 4.4)	0.10
**Operative characteristics**			
Operation duration (h)	4.9 ± 0.4	4.7 ± 0.5	0.30
Perfusion duration (min)	65.9 ± 11.4	61.7 ± 9.9	0.17

Abbreviations: BMI, Body Mass Index; CRP, C-reactive protein; ESR, erythrocyte sedimentation rate; WBC, white blood cell.

^a^ Values are expressed as mean ± SD or No. (%).

^b^ Values subjected to comparison through the application of an independent *t*-test (or a Mann-Whitney U-test in instances where the data exhibited a skewed distribution) or a chi-square test.

**Table 2.** Body Weight and Physical Activity Level in the Two Groups ^a^

Variables	Placebo (n = 25)	Zinc-Vitamin E (n = 27)	P ^b^
**Physical activity (METs/day)**			
Baseline	30.7 ± 10.1	31.8 ± 9.9	0.699
End of study	23.9 ± 1.9	24.4 ± 1.1	0.268
**Body weight (kg)**			
Baseline	64.3 ± 11.9	71.2 ± 12.4	0.049
End of study	61.4 ± 10.9	69.6 ± 12.0	0.004
**Postoperative weight loss (kg)**	2.9 ± 2.9	1.6 ± 1.7	0.047
** Postoperative weight loss (%)**	4.3 ± 3.9	2.1 ± 2.4	0.024

^a^ Values are expressed mean ± SD.

^b^ The P-values for between-group comparisons at the end of the study are calculated using univariate ANOVA, with the baseline value adjusted for.

Table 3 presents the food intake of the participants. In comparison to the baseline data, the placebo group exhibited a reduction in energy intake at the three-week mark following surgery. Nevertheless, no significant difference was observed in energy intake between the two groups. Protein intake and other macronutrients did not differ between the two groups at baseline. Protein intake in both groups was lower at week 3 after surgery than at the beginning of the study, but there was no difference between the two groups.

**Table 3.** Food Intake of Patients in Two Groups at Baseline and 3 Weeks After Surgery ^a^

Variables	Placebo (n = 25)	Zinc-Vitamin E (n = 27)	P ^b^
**Energy (kcal)**			
Baseline	2056.9 ± 445.3	2044.0 ± 430.8	0.916
End of study	1858.5 ± 416.8 ^c^	1959.2 ± 289.7	0.314
**Carbohydrate (g)**			
Baseline	292.1 ± 76.5	286.7 ± 73.1	0.794
End of study	266.8 ± 63.5	279.8 ± 49.8	0.417
**Protein (g)**			
Baseline	92.70 ± 26.2	89.49 ± 22.7	0.638
End of study	73.4 ± 19.9 ^c^	79.34 ± 15.7 ^c^	0.240
**Fat (g) **			
Baseline	59.34 ± 17.8	61.07 ± 15.7	0.712
End of study	57.85 ± 16.4	60.70 ± 13.1	0.492
**Dietary fiber (g)**			
Baseline	13.2 ± 8.4	11.4 ± 5.7	0.345
End of study	14.9 ± 7.1	14.4 ± 6.1	0.798

^a^ Values are expressed as mean ± SD.

^b^ Data were compared using independent sample *t*-test or Mann-Whitney U-test.

^c^ Indicates a significant difference compared to baseline (P < 0.05). The paired samples t-test or Wilcoxon test were employed to assess within-group changes.

Baseline plasma zinc levels were comparable between the two groups at baseline (59.1 ± 19.2 µg/dL in the placebo group and 59.8 ± 16.9 µg/dL in the zinc-vitamin E group, respectively). However, plasma zinc levels were found to be higher in the zinc-vitamin E group on day 3 after surgery (27.24 ± 8.8 µg/dL in the placebo group compared to 38.6 ± 8.8 µg/dL in the zinc-vitamin E group). The zinc-vitamin E group exhibited a significantly higher plasma zinc level at week 3 after surgery compared to the placebo group (71.2 ± 21.3 and 96.7 ± 17.7 µg/dL in the placebo and zinc-vitamin E groups, respectively).

**Figure 2.**
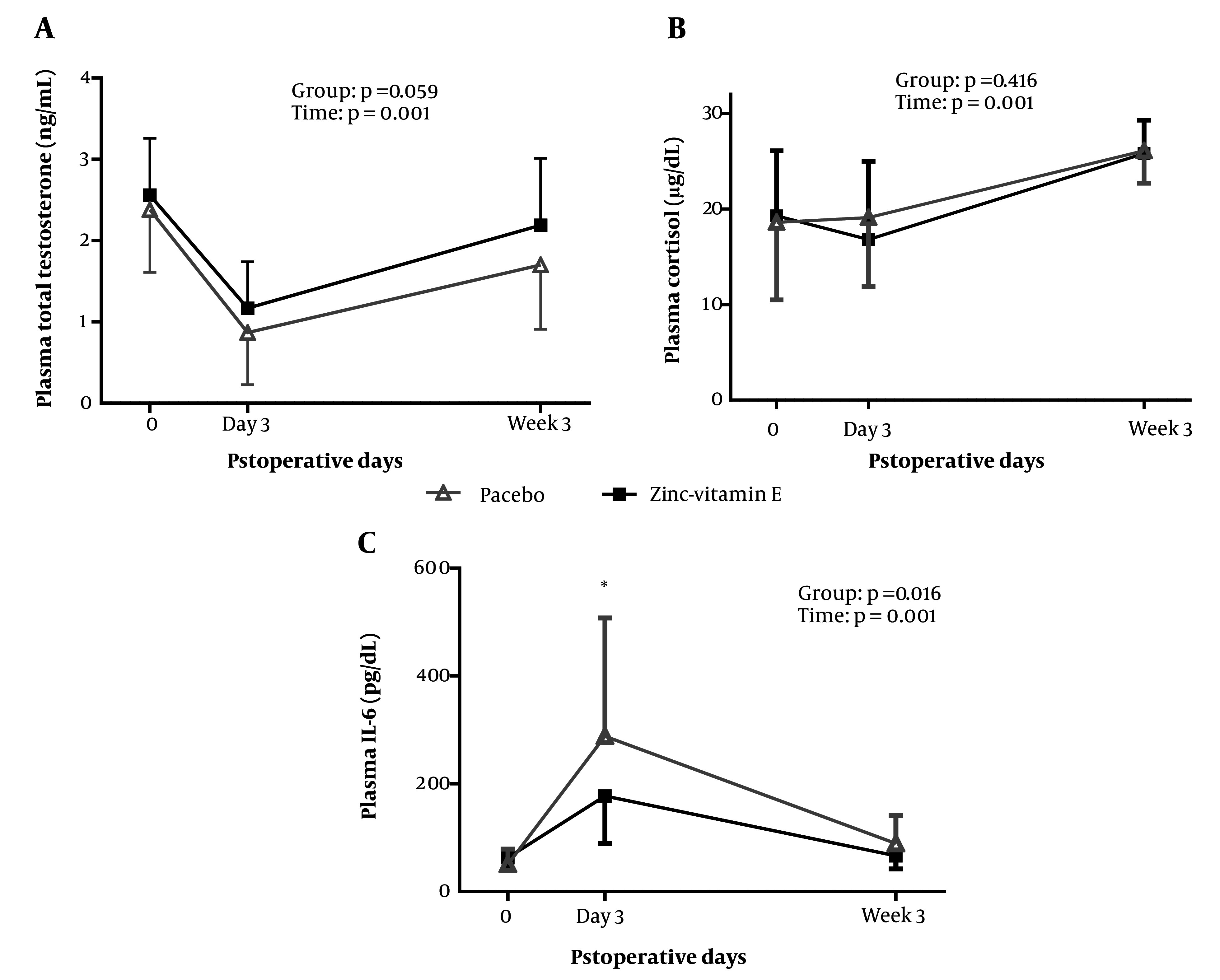
Plasma levels of testosterone, cortisol, and IL-6 at baseline and after surgery in the two groups. The plotted values represent the mean, while the error bars indicate the standard deviation. The treatment effect (group) and changes over time (time) were analyzed using a repeated-measures ANOVA, adjusted for the respective baseline values and body weight. Between-group comparisons on days 3 and week 3 after surgery were analyzed using a univariate ANOVA, adjusted for the respective baseline values and body weight. *P < 0.05. IL-6, interleukin 6.

Figure 2 depicts the alterations in plasma total testosterone. Although plasma testosterone concentrations in the zinc-vitamin E group were relatively higher than in the placebo group, a repeated-measures ANOVA demonstrated that there was no significant difference between the two groups (P = 0.059). There was no significant difference between the two groups in plasma cortisol levels on day 3 and week 3 post-surgery.

**Figure 3.**
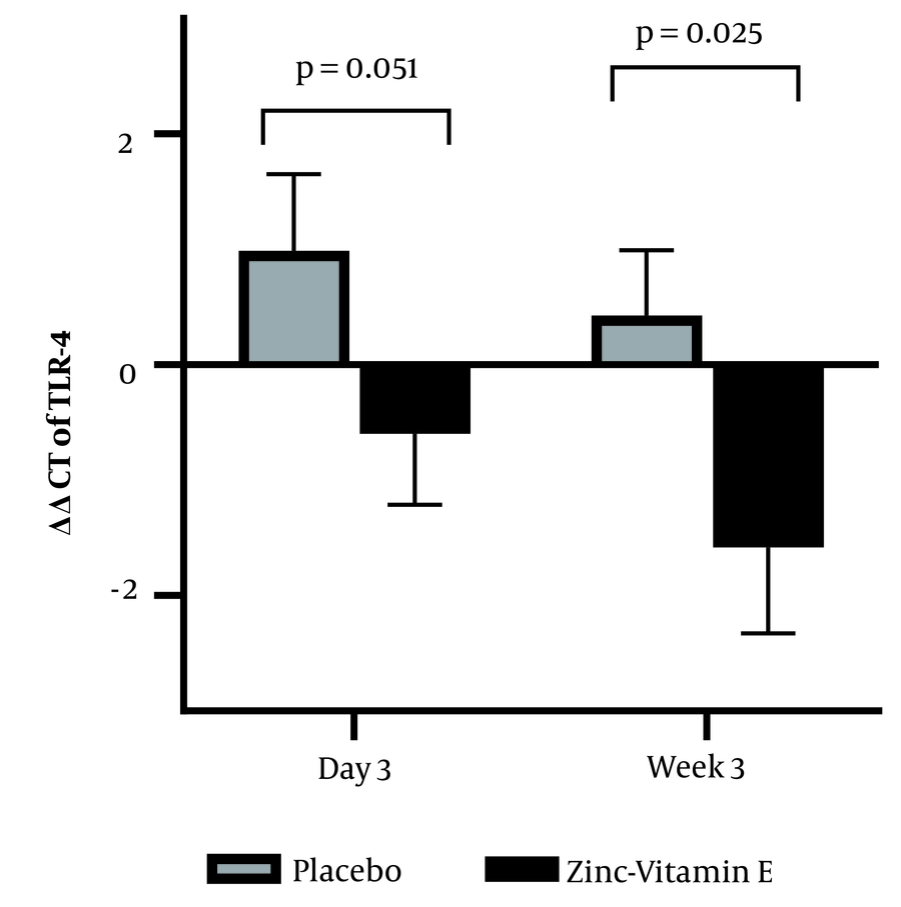
Effect of perioperative zinc and vitamin E supplementation on the mRNA expression of leukocyte toll-like receptor-4. The bars represent the mean, while the error bars represent the standard error of the mean. The ΔCt values calculated on day 3 or week 3 post-surgery were subtracted from the baseline ΔCt values to obtain the ΔΔCt values (ΔΔCt = ΔCt at baseline − ΔCt on postoperative day 3 or week 3). A higher value indicates a higher level of gene expression. The data were analyzed using ANOVA, with adjustments made for body weight.

A repeated-measures ANOVA revealed that plasma IL-6 levels were lower in the zinc-vitamin E supplementation group (P = 0.016). Group comparison demonstrated that plasma IL-6 levels were decreased on postoperative day 3 (P = 0.025), but no significant effect was observed at 3 weeks after surgery (P = 0.091). Moreover, the qPCR results (Figure 3) demonstrated a reduction in TLR-4 expression in white blood cells when zinc-vitamin E was administered compared to the placebo. This was observed on both day 3 (P = 0.051) and week 3 (P = 0.025) after surgery.

Correlation analyses revealed that on the third day following surgery, the change in plasma zinc concentration was inversely correlated with the change in plasma IL-6 concentration (r = -0.31; P = 0.023). However, three weeks after surgery, the change in plasma zinc concentrations was positively correlated with the change in plasma testosterone levels (r = 0.32; P = 0.025) and inversely correlated with the change in plasma IL-6 concentration (r = -0.39; P = 0.004).

## 5. Discussion

The results of this study indicate that the administration of zinc and vitamin E supplements prior to elective CABG surgery was associated with a reduction in postoperative plasma concentrations of IL-6 and the expression of the TLR-4 gene in white blood cells. However, this intervention did not result in a significant change in plasma testosterone levels.

Both groups exhibited a reduction in body weight following surgery, with the placebo group demonstrating a more pronounced decline. A reduction in appetite and dietary intake may contribute to the observed weight loss. Nevertheless, there was no significant difference in the intake of energy and macronutrients between the two groups. However, in comparison to the baseline data, the placebo group exhibited a reduction in energy intake. It is plausible that this reduction in food intake may have contributed to the greater weight loss observed in the placebo group. Other potential causes of weight loss, such as increased diuresis secondary to improved cardiac function, may also have contributed to the observed results.

Cardiopulmonary bypass is associated with significant stress, resulting in strong inflammatory and hormonal responses. In a pilot study of patients undergoing cardiothoracic surgery, low total testosterone levels were observed in 13 of 13 male patients who remained in intensive care units for more than seven days after cardiac surgery (20). In male patients with severe illness, low testosterone levels were maintained or even exacerbated as the disease progressed (6). In the present study, plasma total testosterone levels were found to be decreased compared to baseline (before surgery) in both groups on day 3 after surgery. However, there was an increase in plasma total testosterone levels on week 3 after surgery. Zinc and vitamin E supplementation had no significant effect on plasma total testosterone concentration compared to the placebo group. However, there was a positive correlation between the change in zinc concentration on week 3 after surgery and the change in plasma testosterone concentration. It has been demonstrated that a reduction in dietary zinc intake is associated with a decline in serum testosterone concentrations in young men (21). However, the results of studies investigating the effect of zinc supplementation on serum testosterone concentration have been inconsistent. The administration of zinc supplementation had no significant effect on serum testosterone levels in healthy male volunteers who engaged in regular exercise (22). However, a recent systematic review concluded that zinc supplementation has a positive effect on testosterone levels (17). Zinc supplementation has been shown to increase circulating testosterone concentrations in pathological conditions where zinc status may be poor. These include sickle-cell anemia (12), hemodialysis (10, 11), and growth retardation (23). It is possible that zinc supplementation may increase low testosterone levels in individuals with zinc deficiency but may not further increase serum testosterone with adequate zinc intake (22). It is also the case that surgical intervention results in a reduction in the concentration of zinc in the bloodstream due to the redistribution of zinc and the subsequent lowering of its plasma concentration (24). The observed positive correlation between changes in plasma zinc and testosterone levels in the present study may be interpreted as indirect evidence that zinc has an effect on testosterone levels in these patients. Nevertheless, due to the limited sample size of the present study, the impact of zinc on the testosterone concentration of CABG patients may be more accurately elucidated in studies with larger sample sizes.

The present study found that the use of a zinc-vitamin E supplement had no significant effect on plasma total cortisol levels. Given that cortisol secretion in the systemic circulation occurs in a pulsatile manner, a more accurate assessment of plasma cortisol concentration may necessitate the use of time series with frequent sampling (25). Previous studies have demonstrated that serum cortisol concentrations increase on the first and second postoperative days in patients undergoing elective CABG (26, 27).

It is well established that CABG surgery induces an inflammatory response. The current study demonstrated that zinc and vitamin E supplementation resulted in a reduction in the level of IL-6. In previous studies, supplementation with either zinc (14) or vitamin E (15) has been demonstrated to decrease the level of IL-6. Given that there was a negative correlation between the change in plasma zinc levels and the change in IL-6 levels, it can be concluded that zinc supplementation has contributed to the reduction of IL-6. The use of a zinc-vitamin E supplement demonstrated a more pronounced effect on the reduction of IL-6 on the third day following surgery than on the third week. This may be attributed to the heightened inflammatory response observed on the third day post-surgery, relative to the third week. One potential mechanism by which zinc and vitamin E may affect inflammation is through the regulation of toll-like receptor expression. This hypothesis is corroborated by the findings of the present study, which demonstrate a reduction in the expression of TLR-4 on white blood cells in the zinc-vitamin E group. It is possible that microbial invasion may contribute to the postoperative inflammatory response. However, it is also possible that endogenous non-infectious danger signals may be released during surgery and cause the activation of innate immunity (28). TLR-4 plays a pivotal role in the activation of innate immunity by recognizing specific molecular patterns present in microbes or endogenous damage-associated molecular patterns. Coronary artery bypass grafting results in the release of an endogenous ligand for TLR-4, such as inducible heat shock protein 70, into the circulation, thereby mediating the inflammatory response (28). The results of our study are consistent with those of an animal study that demonstrated a reduction in intestinal mRNA levels of the TLR-4 gene and its downstream signals associated with inflammation in weaning piglets following zinc oxide supplementation (18). Furthermore, vitamin E has been demonstrated to reduce the mRNA abundance of embryonic TLR-3, TLR-7, and TLR-9 in virus-challenged mice (29). However, in contrast to our findings, another preclinical study reported that zinc and vitamin E did not result in a decrease in TLR-4 mRNA expression in broiler jejunum mucosa (30). Moreover, in the limited clinical studies conducted thus far, zinc supplementation did not result in a reduction in TLR-4 gene expression in neonates (31) or in patients with Behçet's disease (32). The disparate outcomes may be attributable to variations in the study subjects or study protocols.

Although the doses of zinc and vitamin E in the present study were higher than the Dietary Reference Intakes, they were not higher than the Tolerable Upper Intake Levels. Additionally, the recommended dietary intakes are based on healthy individuals, and the amount needed during acute illness may be different. Given the short duration of the current study (3 weeks), no adverse effects were expected to result from the use of the dietary supplements. Therefore, no clinical or laboratory evaluations were conducted in this study to assess the potential for adverse effects. It should be noted that oral vitamin E has been demonstrated to exhibit minimal toxicity in both animal and human studies. Short-term (30-day) vitamin E supplementation in an oral dose of 800 mg (> 800 IU) does not result in adverse effects on hepatic or renal function, hematological status, or intermediary metabolism in healthy older subjects (33). Longer-term studies with higher doses of vitamin E (1200 - 2000 IU/day) in patients who had undergone coronary angioplasty (34) or in patients with Parkinson's disease also reported no side effects (35). One of the principal adverse effects associated with high doses of zinc is the disruption of the absorption of other minerals, particularly copper. Nevertheless, it has been demonstrated that zinc supplementation (30 mg per day) for 14 weeks had no effect on copper and ceruloplasmin status in healthy men (36).

It should be noted that this sub-analysis is subject to several limitations, and therefore, any inferences drawn from it should be made with caution. Firstly, this was a secondary analysis of a subset of patients included in a study that was designed to assess the primary outcome of the length of hospitalization. Consequently, the sample size of this study may not be sufficiently large to detect a significant difference in the examined variables. The use of larger samples may, however, lead to the identification of statistically significant differences between the groups. Nevertheless, the analysis of inflammatory markers was a prespecified secondary analysis of the original study. Secondly, in addition to total testosterone, the assessment of free testosterone and sex-hormone-binding globulin could provide a more accurate reflection of testosterone status. However, the quantity of stored plasma sample was insufficient for the subsequent measurements. Furthermore, it would have been advantageous to determine the plasma vitamin E concentrations. Such data would assist in examining the correlation between changes in vitamin E concentration and the studied parameters, as well as in estimating its relative contribution to the observed results.

### 5.1. Conclusions

The administration of zinc and vitamin E to patients undergoing CABG surgery resulted in a relative improvement in certain indicators related to postoperative inflammation. Although the supplementation had no statistically significant effect on plasma testosterone, the changes in plasma zinc were correlated with testosterone levels. This could provide preliminary evidence for the desirability of conducting additional studies.

## Data Availability

The datasets are available from the corresponding author on reasonable request.

## Clinical Trial Registration Code:

NCT05402826.

## References

[1] Jongman RM, Zijlstra JG, Kok WF, van Harten AE, Mariani MA, Moser J (2014). Off-pump CABG surgery reduces systemic inflammation compared with on-pump surgery but does not change systemic endothelial responses: A prospective randomized study.. Shock..

[2] Spratt DI, Kramer RS, Morton JR, Lucas FL, Becker K, Longcope C (2008). Characterization of a prospective human model for study of the reproductive hormone responses to major illness.. Am J Physiol Endocrinol Metab..

[3] Maggio M, Ceda GP, De Cicco G, Cattadori E, Visioli S, Ablondi F (2005). Acute changes in circulating hormones in older patients with impaired ventricular function undergoing on-pump coronary artery bypass grafting.. J Endocrinol Invest..

[4] Becerra-Diaz M, Song M, Heller N (2020). Androgen and androgen receptors as regulators of monocyte and macrophage biology in the healthy and diseased lung.. Front Immunol..

[5] Rettew JA, Huet-Hudson YM, Marriott I (2008). Testosterone reduces macrophage expression in the mouse of toll-like receptor 4, a trigger for inflammation and innate immunity.. Biol Reprod..

[6] Luppa P, Munker R, Nagel D, Weber M, Engelhardt D (1991). Serum androgens in intensive-care patients: Correlations with clinical findings.. Clin Endocrinol (Oxf)..

[7] Almoosa KF, Gupta A, Pedroza C, Watts NB (2014). Low testosterone levels are frequent in patients with acute respiratory failure and are associated with poor outcomes.. Endocr Pract..

[8] Wright TJ, Dillon EL, Durham WJ, Chamberlain A, Randolph KM, Danesi C (2018). A randomized trial of adjunct testosterone for cancer-related muscle loss in men and women.. J Cachexia Sarcopenia Muscle..

[9] Miyoshi M, Tsujimura A, Miyoshi Y, Uesaka Y, Nozaki T, Shirai M (2023). Low serum zinc concentration is associated with low serum testosterone but not erectile function.. Int J Urol..

[10] Mahajan SK, Abbasi AA, Prasad AS, Rabbani P, Briggs WA, McDonald FD (1982). Effect of oral zinc therapy on gonadal function in hemodialysis patients. A double-blind study.. Ann Intern Med..

[11] Jalali GR, Roozbeh J, Mohammadzadeh A, Sharifian M, Sagheb MM, Hamidian Jahromi A (2010). Impact of oral zinc therapy on the level of sex hormones in male patients on hemodialysis.. Ren Fail..

[12] Prasad AS, Abbasi AA, Rabbani P, DuMouchelle E (1981). Effect of zinc supplementation on serum testosterone level in adult male sickle cell anemia subjects.. Am J Hematol..

[13] Hartman TJ, Dorgan JF, Virtamo J, Tangrea JA, Taylor PR, Albanes D (1999). Association between serum alpha-tocopherol and serum androgens and estrogens in older men.. Nutr Cancer..

[14] Bao B, Prasad AS, Beck FW, Fitzgerald JT, Snell D, Bao GW (2010). Zinc decreases C-reactive protein, lipid peroxidation, and inflammatory cytokines in elderly subjects: A potential implication of zinc as an atheroprotective agent.. Am J Clin Nutr..

[15] Devaraj S, Jialal I (2000). Alpha tocopherol supplementation decreases serum C-reactive protein and monocyte interleukin-6 levels in normal volunteers and type 2 diabetic patients.. Free Radic Biol Med..

[16] Briassoulis G, Briassoulis P, Ilia S, Miliaraki M, Briassouli E (2023). The anti-oxidative, anti-inflammatory, anti-apoptotic, and anti-necroptotic role of zinc in COVID-19 and Sepsis.. Antioxidants (Basel)..

[17] Te L, Liu J, Ma J, Wang S (2023). Correlation between serum zinc and testosterone: A systematic review.. J Trace Elem Med Biol..

[18] Hu CH, Song ZH, Xiao K, Song J, Jiao le F, Ke YL (2014). Zinc oxide influences intestinal integrity, the expressions of genes associated with inflammation and TLR4-myeloid differentiation factor 88 signaling pathways in weanling pigs.. Innate Immun..

[19] Makiabadi E, Nakhaeizadeh R, Soleimani M, Nasrollahzadeh J (2024). Effects of perioperative vitamin E and zinc co-supplementation on systemic inflammation and length of stay following coronary artery bypass graft surgery: A randomized controlled trial.. Eur J Clin Nutr..

[20] Ward CT, Boorman DW, Afshar A, Prabhakar A, Fiza B, Pyronneau LR (2021). A screening tool to detect chronic critically ill cardiac surgery patients at risk for low levels of testosterone and somatomedin c: A prospective observational pilot study.. Cureus..

[21] Hunt CD, Johnson PE, Herbel J, Mullen LK (1992). Effects of dietary zinc depletion on seminal volume and zinc loss, serum testosterone concentrations, and sperm morphology in young men.. Am J Clin Nutr..

[22] Koehler K, Parr MK, Geyer H, Mester J, Schanzer W (2009). Serum testosterone and urinary excretion of steroid hormone metabolites after administration of a high-dose zinc supplement.. Eur J Clin Nutr..

[23] Ghavami-Maibodi SZ, Collipp PJ, Castro-Magana M, Stewart C, Chen SY (1983). Effect of oral zinc supplements on growth, hormonal levels, and zinc in healthy short children.. Ann Nutr Metab..

[24] Hou HT, Xue LG, Zhou JY, Wang SF, Yang Q, He GW (2020). Alteration of plasma trace elements magnesium, copper, zinc, iron and calcium during and after coronary artery bypass grafting surgery.. J Trace Elem Med Biol..

[25] Teblick A, Peeters B, Langouche L, Van den Berghe G (2019). Adrenal function and dysfunction in critically ill patients.. Nat Rev Endocrinol..

[26] Roth-Isigkeit AK, Dibbelt L, Schmucker P (2000). Blood levels of corticosteroid-binding globulin, total cortisol and unbound cortisol in patients undergoing coronary artery bypass grafting surgery with cardiopulmonary bypass.. Steroids..

[27] Roth-Isigkeit A, Dibbelt L, Eichler W, Schumacher J, Schmucker P (2001). Blood levels of atrial natriuretic peptide, endothelin, cortisol and ACTH in patients undergoing coronary artery bypass grafting surgery with cardiopulmonary bypass.. J Endocrinol Invest..

[28] Dybdahl B, Wahba A, Lien E, Flo TH, Waage A, Qureshi N (2002). Inflammatory response after open heart surgery: Release of heat-shock protein 70 and signaling through toll-like receptor-4.. Circulation..

[29] Wu D, Luo XL, Lin Y, Fang ZF, Luo XR, Xu HT (2010). Effects of vitamin E on reproductive protection in pregnant mice infected with pseudorabies virus (PRV) via regulating expression of Toll-like receptors (TLRs) and cytokine balance.. J Nutr Sci Vitaminol (Tokyo)..

[30] Song Z, Lv J, Sheikhahmadi A, Uerlings J, Everaert N (2017). Attenuating effect of zinc and vitamin e on the intestinal oxidative stress induced by silver nanoparticles in broiler chickens.. Biol Trace Elem Res..

[31] Banupriya N, Bhat BV, Vickneshwaran V, Sridhar MG (2020). Effect of zinc supplementation on relative expression of immune response genes in neonates with sepsis: A preliminary study.. Indian J Med Res..

[32] Faghfouri AH, Khabbazi A, Baradaran B, Khajebishak Y, Baghbani E, Noorolyai S (2022). Immunomodulatory and clinical responses to zinc gluconate supplementation in patients with Behcet's disease: A double-blind, randomized placebo-controlled clinical trial.. Clin Nutr..

[33] Meydani SN, Meydani M, Rall LC, Morrow F, Blumberg JB (1994). Assessment of the safety of high-dose, short-term supplementation with vitamin E in healthy older adults.. Am J Clin Nutr..

[34] DeMaio SJ, King S3, Lembo NJ, Roubin GS, Hearn JA, Bhagavan HN (1992). Vitamin E supplementation, plasma lipids and incidence of restenosis after percutaneous transluminal coronary angioplasty (PTCA).. J Am Coll Nutr..

[35] Parkinson Study G (1993). Effects of tocopherol and deprenyl on the progression of disability in early Parkinson's disease.. N Engl J Med..

[36] Bonham M, O'Connor JM, McAnena LB, Walsh PM, Downes CS, Hannigan BM (2003). Zinc supplementation has no effect on lipoprotein metabolism, hemostasis, and putative indices of copper status in healthy men.. Biol Trace Elem Res..

